# Lipomatosis Incidence and Characteristics in an Italian Cohort of Mitochondrial Patients

**DOI:** 10.3389/fneur.2019.00160

**Published:** 2019-02-27

**Authors:** Olimpia Musumeci, Emanuele Barca, Costanza Lamperti, Serenella Servidei, Giacomo Pietro Comi, Maurizio Moggio, Tiziana Mongini, Gabriele Siciliano, Massimiliano Filosto, Elena Pegoraro, Guido Primiano, Dario Ronchi, Liliana Vercelli, Daniele Orsucci, Luca Bello, Massimo Zeviani, Michelangelo Mancuso, Antonio Toscano

**Affiliations:** ^1^Department of Clinical and Experimental Medicine, UOC Neurologia e Malattie Neuromuscolari, University of Messina, Messina, Italy; ^2^Department of Neurology, Columbia University Medical Center, New York, NY, United States; ^3^UO of Medical Genetics and Neurogenetics, The Foundation “Carlo Besta” Institute of Neurology-IRCCS, Milan, Italy; ^4^UOC Neurofisiopatologia, Fondazione Policlinico Universitario A. Gemelli IRCCS, Istituto di Neurologia Università Cattolica del Sacro Cuore, Rome, Italy; ^5^Neurology Unit, Neuroscience Section, Department of Pathophysiology and Transplantation, Dino Ferrari Centre, IRCCS Foundation Ca' Granda Ospedale Maggiore Policlinico, University of Milan, Milan, Italy; ^6^Neuromuscular and Rare Diseases Unit, Department of Neuroscience, Fondazione IRCCS Ca' Granda, Ospedale Maggiore Policlinico, Milan, Italy; ^7^Department of Neurosciences Rita Levi Montalcini, University of Torino, Torino, Italy; ^8^Department of Clinical and Experimental Medicine, Neurological Institute, University of Pisa, Pisa, Italy; ^9^Unit of Neurology, Center for Neuromuscular Diseases, ASST Spedali Civili and University of Brescia, Brescia, Italy; ^10^Department of Neurosciences, University of Padova, Padova, Italy; ^11^Mitochondrial Biology Unit, Medical Research Council, Cambridge, United Kingdom

**Keywords:** multiple symmetrical lipomatosis, MERRF, mitochondrial myopathy, madelung's disease, brown fat

## Abstract

Lipomas have often been associated with mtDNA mutations and were mainly observed in patients with mutation in mitochondrial tRNAlysine which is also the most frequent mutation associated with MERRF. Up to date, no systematic studies have been developed in order to assess the incidence of lipomas in large cohorts of mitochondrial patients.The aim of this study is to analyze the incidence and characteristics of lipomas among an Italian cohort of patients with mitochondrial diseases. A retrospective, database-based study (Nation-wide Italian Collaborative Network of Mitochondrial Diseases) of patients with lipomas was performed. A total of 22 (1.7%) patients with lipomas have been identified among the 1,300 mitochondrial patients, enrolled in the Italian database. In about 18% multiple systemic lipomatosis (MSL) was the only clinical manifestation; 54% of patients showed a classical MERRF syndrome. Myopathy, alone or in association with other symptoms, was found in 27% of patients. Lactate was elevated in all the 12 patients in which was measured. Muscle biopsy was available in 18/22 patients: in all of them mitochondrial abnormalities were present. Eighty six percent had mutations in mtDNA coding for tRNA lysine. In most of patients, lipomas were localized along the cervical-cranial-thoracic region. In 68% of the patients were distributed symmetrically. Only two patients had lipomas in a single anatomical site (1 in right arm and 1 in gluteus maximum). MSL is often overlooked by clinicians in patients with mitochondrial diseases where the clinical picture could be dominated by a severe multi-systemic involvement. Our data confirmed that MSL is a rare sign of mitochondrial disease with a strong association between multiple lipomas and lysine tRNA mutations. MSL could be considered, even if rare, a red flag for mitochondrial disorders, even in patients with an apparently isolated MSL.

## Introduction

Multiple systemic lipomatosis (MSL) is a rare disorder involving adipose tissue and characterized, clinically, by the development of non-encapsulated lipomas usually distributed in the cervical–cranial–thoracic region (1–3). Since the first description, MSL was differently named referring to scientists who described some peculiar clinical features of the disease as Brodie syndrome or Madelung‘s disease or Launois–Bensaude disease (1–3).

Lipomatous masses often occur in the third/fourth decade of life. MSL is largely prevalent among males and it has been correlated with high alcohol intake (4, 5). Lipomas ‘distribution is prevalent along the midline, sparing distal portion of the limbs. Visceral sites can also be involved. The course of MSL is usually benign although some authors reported a higher mortality comparing with the general population. Respiratory airways obstruction by cervical lipomatous masses is a common serious complication (4, 6).

The pathogenesis of lipoma formation is unclear but sporadic observations have linked MSL with mitochondrial dysfunction (7).

In about 28% of MSL patients, mitochondrial alterations (e.g., ragged red fibers –RRF- and COX negative fibers) were noted in muscle biopsies (8). Mitochondrial DNA mutations have been recurrently diagnosed in MSL patients (about 16% of tested patients) being the mitochondrial lysine tRNA (m. 8344 A>G, known to be associated with Myoclonic-Epilepsy with Ragged Red Fibers –MERRF- syndrome) (9–12). In addition recently, MSL has been observed in a family with multiple deletion of mitochondrial DNA and a mutation in Mitofusin 2 (13).

The pathogenesis of lipomas in MSL seems to be due to alteration of brown fat tissue growth regulation, this hypothesis is supported by the typical anatomic distribution of the masses and from results of morphological studies. Lipomatous masses are indeed located along the midline of the body, following the distribution of brown adipose tissue (BAT) present in newborns (14). BAT, contrary to the white adipose tissue, produces heat because of an overexpression of UCP1 (Uncoupling Protein 1) that is responsible for the uncoupling of the oxidative phosphorylation from ATP production (15). MSL tissues show overexpression of UCP1, confirming the it may be originated from BAT (16).

While mitochondrial function in patients with idiopathic MSL has been systematically studied (9), the prevalence of MSL in mitochondrial patients has been only evaluated in few cases reports. Our study aims to explore the presence of this syndrome in a large cohort of Italian patients with mitochondrial diseases.

## Materials and Methods

We reviewed data of 1,300 patients reported in the database of the Nation-wide Italian Collaborative Network of Mitochondrial Diseases searching for the presence of lipomatosis as clinical feature. Aggregated data were using to evaluate: (1) incidence of lipomas among the population of Italian mitochondrial patients, (2) molecular defects present in patients with MSL, (3) clinical characteristics of lipomas (anatomical distribution, degree of symmetry), (4) clinical phenotypes of patients (MERRF, chronic external ophthalmoplegia—cPEO etc.), (5) correlation between MSL and other biochemical markers.

This was a retrospective study. The clinical features were extrapolated from the web-based database in which every item, considered relevant for mitochondrial disorders in a previous consensus phase among all involved centers, was evaluated according to the presence or not (“yes or no”).

MSL severity has been graded considering the number of anatomical region involved and the symmetry. Data were expressed as mean ± Standard deviation (SD).

The local ethical committees of all involved centers have approved the database establishment and its use for scientific purpose.

All enrolled patients, in accordance with the ethical standards of the 1964 Declaration of Helsinki, have provided informed consent.

Linear correlation was calculated using Graph pad Prism 6 software, statistical comparisons were tested using Pearson r test (for correlations) and Student *t*-test (for means) with alpha < 0.05.

## Results

### MSL Incidence in the Database and Demographic Characteristics

Among the 1,300 patients of the Italian registry, a total of 22 patients from 19 independent families with MSL have been identified, representing the 1.7% of all subjects of the database. Fourteen MSL patients were female (63%) and 8 males (37%). Age of onset was after the second decade in most of the patients with MSL apart from two sisters with encephalomyopathy with a very early onset in the first year of life. No differences have been observed between mean age at onset in male vs. female subjects (Female 34.5 years vs. Male 35.7 years).

### Clinical Characteristics of MSL Patients

The majority of subjects with MSL showed a variety of signs and symptoms but only in four subjects (18%) it was an isolated manifestation in a mean follow-up period of about 15 years. About these four, two were relatives of affected individuals with other clinical manifestations whereas two were primarily investigated for a mitochondrial disorder because of lipomas. Twelve subjects (54.4%) had a diagnosis of MERRF syndrome; six patients (27%) had a clear myopathy with muscle wasting and weakness (Table 1).

**Table 1:** Clinical and laboratory features.

**Pt**	**Age at onset**	**Disease duration**	**Clinical phenotypes**	**Sites**	**Multiple**	**Symmetric**	**Muscle biopsy**	**mtDNA mutation**
1	28	25	MERRF	Neck	Yes	No	ND	8344A>G
2	55	13	Myopathy	Neck , trunk	Yes	Yes	ND	8344A>G
3	45	20	MERRF	Neck	No	Yes	RRF	8344A>G
4	40	31	Lipomatosis	Neck, trunk	Yes	No	RRF	8344A>G
5	42	19	MERRF	Neck, trunk, shoulders, limbs	Yes	Yes	RRF, COX -	8344A>G
6	40	18	Ataxia, spastic paraparesis, optic atrophy	Neck, shoulders, limbs	Yes	Yes	RRF, COX-, dystrophic features	Multiple deletions
7	50	10	Encephalomyopathy	Neck, shoulders, limbs	Yes	Yes	RRF, COX-	Multiple deletions
8	1	33	Encephalomyopathy	Neck	No	Yes	RRF, COX-	8363G>A
9	1	33	MERRF	Neck	No	Yes	RRF, COX-	8363G>A
10	66	5	Lipomatosis, polineuropathy, eyelid ptosis	Neck	Yes	Yes	RRF, COX-	8344A>G
11	54	19	cPEO, ataxia	Neck	Yes	Yes	Fiber atrophy and subsarcolemmal rims at SDH stain	Multiple deletions
12	20	35	cPEO, myopathy	Right gluteus maximus (intramuscular)	No	No	RRF, COX-, dystrophic features	8344A>G
13	30	35	MERRF	Right arm	No	No	RRF, COX-	8344A>G
14	30	16	MERRF	Neck and back	Yes	Yes	RRF, COX-	8344A>G
15	50	20	MERRF	Neck and back	Yes	Yes	ND	8344A>G
16	25	24	MERRF	Neck, back, shoulder, arm, mediastinum	Yes	Yes	RRF, COX-	8344A>G
17	40	3	MERRF	Neck and back	Yes	Yes	RRF, COX-	8344A>G
18	38	3	MERRF	Neck and back, right shoulder and arm	Yes	No	RRF, COX-	8344A>G
19	8	27	KSS	Left forearm	Yes	No	RRF, COX-	8344A>G
20	35	4	MERRF	Neck and back, right shoulder and arm	Yes	Yes	RRF, COX-	8344A>G
21	40	20	Isolated lipomas	Left forearm and abdomen	Yes	No	ND	8344A>G
22	30	20	MERRF	Neck and back	Yes	Yes	RRF, COX-	8344A>G

*cPEO, chronic progressive external ophthalmoplegia; MERRF, mitochondrial encephalomyopathy with ragged red fibers; KSS, Kearn-Sayre syndrome*.

In two subjects MSL was associated with CPEO. Two patients had cerebellar ataxia whereas peripheral neuropathy was observed in five individuals.

Chronic high alcohol intake, diabetes, or dyslipidemia were not present in any of the individuals with MSL.

Plasma lactate dosage was available in 12 subjects: it was elevated in all of them of whom 11 had high lactate level at rest (ranging from 2.7 mEq/l to 4.8 mEq /l, reference values: 0.5–1.5 mEq/l) while only in one subject it was elevated after exercise.

### Muscle Morphological and Biochemical Features

Muscle biopsy was performed in 18 subjects out of 22. RRF and COX negative fibers were reported in 15 out of 18 (83.2%), two patients had only RRF, and one (with cPEO) had only type II fiber atrophy and increased sub-sarcolemma rims at SDH staining. Dystrophic features were reported in two (one with myopathy, and the other with cerebellar ataxia and deafness). Biochemical activities of the respiratory chain enzymes were assessed in 8 individuals; in 3 of them, a reduction of COX activity was reported, complex II+III were reduced in other 3 subjects, one individual had a reduction in complex I activity and another one multiple enzymes defects.

### Genetic Description of MSL Cohort

Genetic data showed that most of the patients with MSL (19 patients, 86%) harbored point mutations in mitochondrial DNA (mtDNA) gene coding for lysine tRNA (tRNA^Lys^). Among them, 17 (77% of the MSL cohort) had the m.8344 A > G and two (9%) carried the m.8363G>A. Data on heteroplasmy levels (expressed as percentage of mutated over wild type mtDNA) were recorded in blood in 8 patients, in muscle in 7 and in urine sediment in 6. Mean mutation load was lower in blood (62.2 ± 17.2), and slightly higher in muscle (88 ± 6%) and urine sediment (69 ± 26%).

Multiple deletions of mtDNA were recorded in three unrelated patients. The genes associated with mtDNA maintenance (ANT1, POLG1, dGK, and Twinkle) as well as MFN2 have been sequenced but no causative mutations were detected.

### Lipomas Distribution

**Figure 1:**
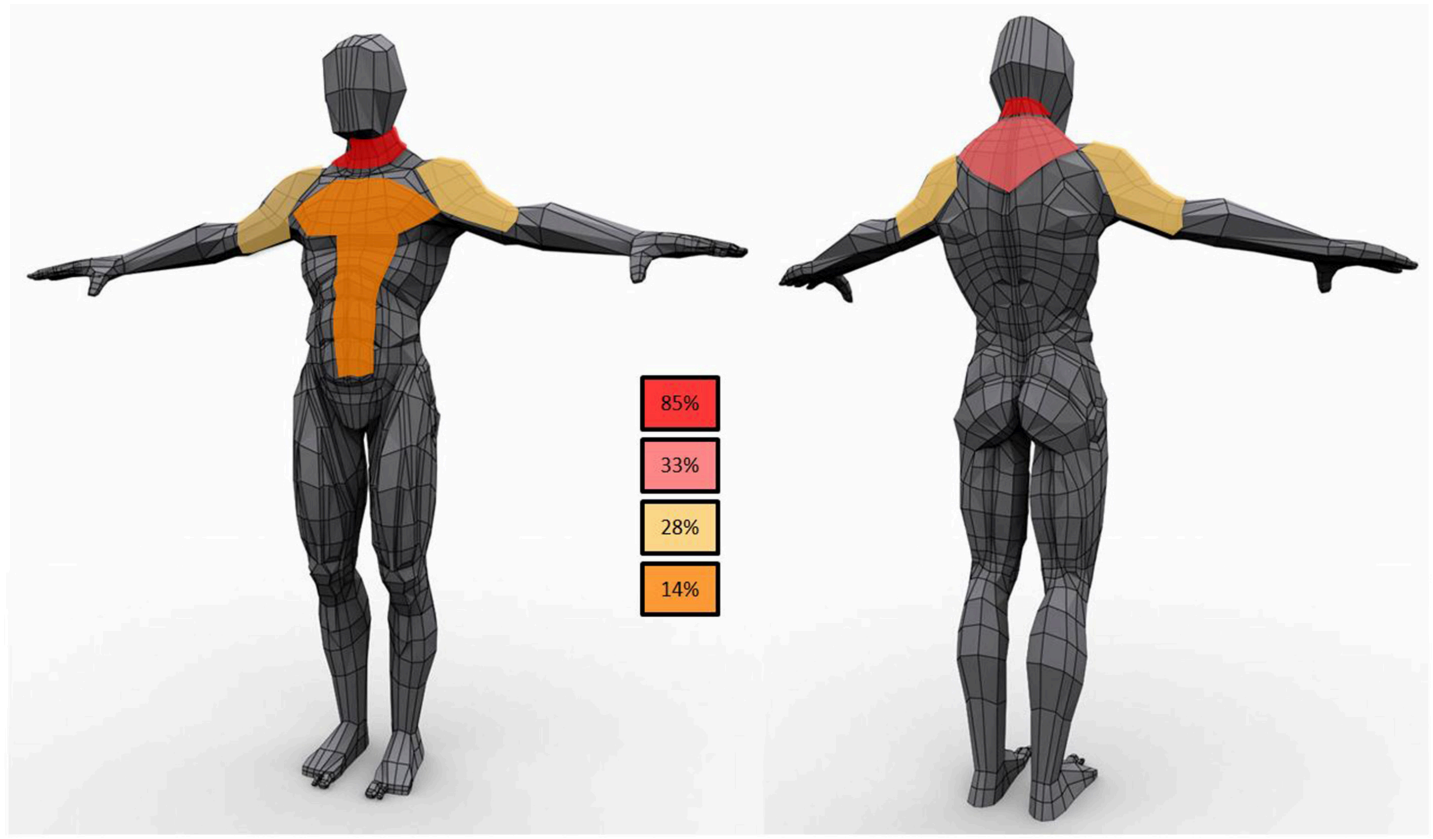
Schematic representation of lipomas distribution in the study population. In the boxes the frequency of observations in the cohort.

Lipomas were distributed symmetrically in 15 patients (68%) (Figure 1); five subjects had lipomas in a single site (3 in the neck, 1 in right arm and 1 in gluteus maximum) whereas multiple involvement was observed in 77% of the cohort with the neck region as the most affected area (82%) followed by proximal upper limb and the posterior part of the shoulders (41 and 27%, respectively). Lipomas were reported in the back in 45% subjects; abdominal wall was affected in 23% whereas mediastinum was involved in two individuals.

## Discussion

MSL was firstly described in the late Nineteenth century and since then, many studies investigated the clinical features and long-term follow-up of MSL patients (4). Alteration in mitochondrial function and MSL have been correlated in several reports of single patients (4). However, no systematic studies have been conducted to assess MSL prevalence and the associated clinical features in large cohorts of mitochondrial patients.

In this study we investigate the prevalence and the characteristics of MSL in the Italian cohort of mitochondrial patients. Among the subjects enrolled in the registry, lipomas were reported in 22 (1.7%), confirming that MSL is a rare disorder even in this specific population. In contrast with literature data obtained in patients with MSL (4) we observed a higher presence of MSL in females than males. Mean age of symptoms onset was overlapping with the one reported in MERRF syndrome (17, 18). Among the all cohort of subjects with the m.8344A > G variant in the Italian registry, the presence of lipomas was reported in 30%. Patients with MERRF + MSL resulted more severely affected than MERRF patients.

MSL can be the only clinical manifestation of a mitochondrial disorder (18% in this cohort). We observed a high association of myopathy (alone or with other symptoms), probably as part of a spectrum of the m.8344A>G severity.

Lactic acid is increased in patients with defect of oxidative phosphorylation, and it is used as marker for mitochondrial diseases, even though it lacks of good specificity and sensitivity (19). Interestingly, all the 12 tested patients have mild to moderate lactic acidosis. This evidence is interesting and may point out to the biochemical properties of MSL overlapping mitochondrial dysfunction (20) and metabolic abnormalities typically of tumorous tissues.

Regarding anatomical distribution, although lipomas were symmetric in the majority of the subjects, in one third of the subjects was not so. This observation, differs from the literature data (4), and suggests that asymmetry does not rule out the diagnosis of lipomatosis in mitochondrial diseases. The pattern of MSL development is similar to the reported distribution for MSL with lipomas along the midline with neck and shoulders very frequently involved.

In conclusion, for the first time, this study focuses on a rare and poorly investigated manifestation of mitochondrial disease. In patients with mitochondrial diseases, MSL can be easily overlooked because other clinical picture usually dominate the clinical course. Our data have demonstrated that MSL is a rare sign of mitochondrial disease. Identification of MSL is however important because it could be considered, a *red flag* for mitochondrial disorders thus guiding the diagnosis. Moreover, our data warns to consider to pursue mitochondrial DNA analysis even in patients with isolated MSL since it can represent the only manifestation of a pathogenic mtDNA mutations. Comparing our results with published data from the literature, we identified some differences that can help to recognize patient with MSL who deserve mitochondrial disease investigation: mitochondrial MSL (1) is more frequent in women, with no history of alcohol abuse, (2) is often associated with muscle involvement and (3) can be asymmetric. The association of MSL and elevated lactate level strongly suggests a mitochondrial disorder.

Our cohort study presents the typical limitations of all retrospective studies, and probably underestimates the real prevalence of MSL in patients with mitochondrial diseases, being a sign that sometimes could be missed when not actively searched, especially if visceral. However, similar multicenter efforts are needed and strongly encouraged for rare disorders such as mitochondrial diseases, and may represent the basis for more rigorous longitudinal studies.

## Author Contributions

OM had full access to all the data in the study and takes responsibility for the integrity of the data and accuracy of data analysis. OM, EB, AT, and MiM contributed to study design. OM, EB, CL, SS, GC, MaM, MF, GS, and MZ contributed to data collection. OM, EB, MiM, and AT drafted the manuscript. TM, GP, DR, EP, LB, LV, and DO provided clinical informations. EB performed statistical analysis. All authors read and approved the final manuscript.

### Conflict of Interest Statement

The authors declare that the research was conducted in the absence of any commercial or financial relationships that could be construed as a potential conflict of interest.
